# 5-Chloro-7-methyl-2-phenyl-3-phenyl­sulfinyl-1-benzofuran

**DOI:** 10.1107/S1600536809024763

**Published:** 2009-07-04

**Authors:** Hong Dae Choi, Pil Ja Seo, Byeng Wha Son, Uk Lee

**Affiliations:** a Department of Chemistry, Dongeui University, San 24 Kaya-dong Busanjin-gu, Busan 614-714, Republic of Korea; b Department of Chemistry, Pukyong National University, 599-1 Daeyeon 3-dong, Nam-gu, Busan 608-737, Republic of Korea

## Abstract

In the title compound, C_21_H_15_ClO_2_S, the O atom and the phenyl group of the phenyl­sulfinyl substituent lie on opposite sides of the plane of the benzofuran fragment; the phenyl ring is almost perpendicular to this plane [82.24 (7)°]. The phenyl ring in the 2-position is rotated out of the benzofuran plane, making a dihedral angle of 11.50 (9)°. The crystal structure is stabilized by inter­molecular C—H⋯O and C—H⋯Cl inter­actions. In addition, the stacked mol­ecules exhibit an inter­molecular S⋯O inter­action [3.327 (2) Å] involving the sulfinyl groups.

## Related literature

For the crystal structures of similar 5-chloro-1-benzofuran derivatives, see: Choi *et al.* (2007 ▶, 2008*a* ▶). For details of sulfin­yl–sulfinyl inter­actions, see: Choi *et al.* (2008*b* ▶). For a review of carbon­yl–carbonyl inter­actions, see: Allen *et al.* (1998 ▶).
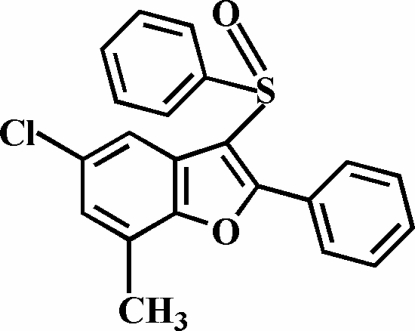


## Experimental

### 

#### Crystal data

C_21_H_15_ClO_2_S
*M* *_r_* = 366.84
Triclinic, 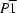

*a* = 8.224 (1) Å
*b* = 10.169 (1) Å
*c* = 11.083 (2) Å
α = 68.771 (2)°
β = 78.050 (2)°
γ = 81.483 (2)°
*V* = 842.5 (2) Å^3^
*Z* = 2
Mo *K*α radiation
μ = 0.36 mm^−1^
*T* = 273 K
0.35 × 0.20 × 0.20 mm

#### Data collection

Bruker SMART CCD diffractometer
Absorption correction: multi-scan (*SADABS*; Sheldrick, 1999 ▶) *T*_min_ = 0.910, *T*_max_ = 0.932
6585 measured reflections
3270 independent reflections
2650 reflections with *I* > 2σ(*I*)
*R*_int_ = 0.018

#### Refinement

*R*[*F* ^2^ > 2σ(*F* ^2^)] = 0.038
*wR*(*F* ^2^) = 0.107
*S* = 1.06
3270 reflections
227 parameters
H-atom parameters constrained
Δρ_max_ = 0.49 e Å^−3^
Δρ_min_ = −0.45 e Å^−3^

### 

Data collection: *SMART* (Bruker, 2001 ▶); cell refinement: *SAINT* (Bruker, 2001 ▶); data reduction: *SAINT*; program(s) used to solve structure: *SHELXS97* (Sheldrick, 2008 ▶); program(s) used to refine structure: *SHELXL97* (Sheldrick, 2008 ▶); molecular graphics: *ORTEP-3* (Farrugia, 1997 ▶) and *DIAMOND* (Brandenburg, 1998 ▶); software used to prepare material for publication: *SHELXL97*.

## Supplementary Material

Crystal structure: contains datablocks global, I. DOI: 10.1107/S1600536809024763/ng2607sup1.cif

Structure factors: contains datablocks I. DOI: 10.1107/S1600536809024763/ng2607Isup2.hkl

Additional supplementary materials:  crystallographic information; 3D view; checkCIF report

## Figures and Tables

**Table 1:** Hydrogen-bond geometry (Å, °)

*D*—H⋯*A*	*D*—H	H⋯*A*	*D*⋯*A*	*D*—H⋯*A*
C19—H19⋯Cl^i^	0.93	2.78	3.653 (3)	157
C20—H20⋯O2^ii^	0.93	2.47	3.261 (3)	144

Symmetry codes: (i) 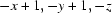; (ii) 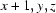.

**Fig. 1.**
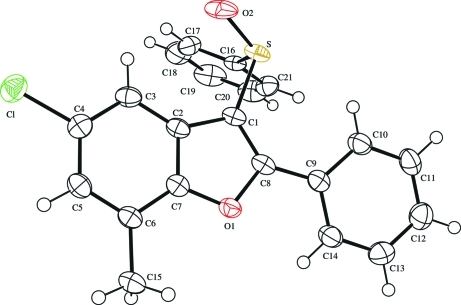
The molecular structure of the title compound with the atom numbering scheme. Displacement ellipsoids are drawn at the 50% probability level. H atoms are presented as a small spheres of arbitrary radius.

**Fig. 2.**
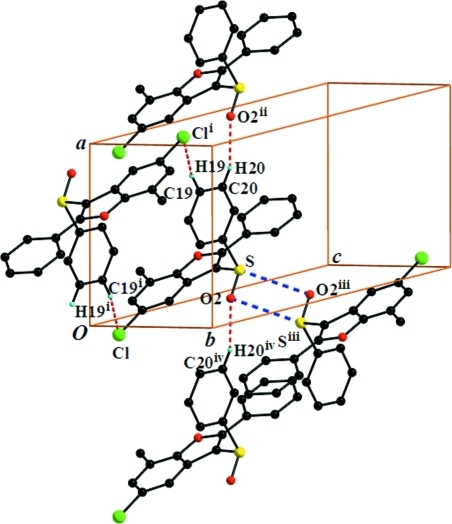
C–H···O, C–H···Cl, and S···O interactions (dotted lines) in the title compound. [Symmetry code: (i) -x + 1, -y + 1, -z; (ii) x + 1, y, z; (iii) -x, -y + 1, -z + 1; (iv) -1 +x, y, z.]

## supplementary crystallographic information

### Comment

This work is related to our communications on the synthesis and structures of 5-chloro-1-benzofuran analogues, *viz*. 5-chloro-3-methylsulfinyl-2-phenyl-1-benzofuran (Choi *et al.*, 2007) and 5-chloro-2-methyl-3-phenylsulfonyl-1-benzofuran (Choi *et al.*, 2008*a*). Here we report the crystal structure of the title compound (I), 5-chloro-7-methyl-2-phenyl-3-phenylsulfinyl-1-benzofuran (Fig. 1).

The benzofuran unit is essentially planar, with a mean deviation of 0.011 (2) Å from the least-squares plane defined by the nine constituent atoms. The dihedral angle in (I) formed by the planes of the benzofuran and the 2-phenyl rings is 11.90 (9)°, and the phenyl ring (C16-C21) with 82.24 (7)° lies toward the benzofuran plane. The crystal packing (Fig. 2) is stabilized by intermolecular C–H···O and C–H···Cl interactions; the first between the phenyl H atom of the phenylsulfinyl substituent and the oxygen of the S═O unit, with a C20–H20···O2^ii^, the second between the phenyl H atom of the phenylsulfinyl substituent and the chlorine of the benzofuran ring, with a C19–H19···Cl^i^, respectively (Table 1 and Fig. 2). In addition, the crystal packing (Fig. 2) exhibits a sulfinyl-sulfinyl interaction (Choi *et al.*, 2008*b*) interpreted as similar to a type-II carbonyl-carbonyl interaction (Allen *et al.*, 1998), with S···O2^iii^ and O2···S^iii^ distance of 3.327 (2) Å.

### Experimental

The 77% 3-chloroperoxybenzoic acid (247 mg, 1.1 mmol) was added in small portions to a stirred solution of 5-chloro-7-methyl-2-phenyl-3-phenylsulfanyl-1-benzofuran (351 mg, 1.0 mmol) in dichloromethane (30 mL) at 273 K. After being stirred at room temperature for 3h, the mixture was washed with saturated sodium bicarbonate solution and the organic layer was separated, dried over magnesium sulfate, filtered and concentrated in vacuum. The residue was purified by column chromatography (hexane-ethyl acetate, 2:1 v/v) to afford the title compound as a colorless solid [yield 76%, m.p. 462-463 K; *R*_f_ = 0.7 (hexane-ethyl acetate, 2:1 v/v)]. Single crystals suitable for X-ray diffraction were prepared by slow evaporation of a solution of the title compound in ethyl acetate at room temperature.

### Refinement

All H atoms were positioned geometrically and refined using a riding model, with C–H = 0.93 Å for aromatic H atoms and 0.96 Å for methyl H atoms, respectively, and with *U*iso(H) = 1.2*U*eq(C) for aromatic H atoms and 1.5 *U*eq(C) for methyl H atoms, respectively.

### Crystal data

**Table:**

C_21_H_15_ClO_2_S	*Z* = 2
*M**_r_* = 366.84	*F*(000) = 380
Triclinic, *P*1	*D*_x_ = 1.446 Mg m^−^^3^
Hall symbol: -P 1	Mo *K*α radiation, λ = 0.71073 Å
*a* = 8.224 (1) Å	Cell parameters from 3597 reflections
*b* = 10.169 (1) Å	θ = 2.4–27.4°
*c* = 11.083 (2) Å	µ = 0.36 mm^−^^1^
α = 68.771 (2)°	*T* = 273 K
β = 78.050 (2)°	Block, colourless
γ = 81.483 (2)°	0.35 × 0.20 × 0.20 mm
*V* = 842.5 (2) Å^3^	

### Data collection

**Table:**

Bruker SMART CCD diffractometer	3270 independent reflections
Radiation source: fine-focus sealed tube	2650 reflections with *I* > 2σ(*I*)
graphite	*R*_int_ = 0.018
Detector resolution: 10.0 pixels mm^-1^	θ_max_ = 26.0°, θ_min_ = 2.0°
φ and ω scans	*h* = −10→10
Absorption correction: multi-scan (*SADABS*; Sheldrick, 1999)	*k* = −12→12
*T*_min_ = 0.910, *T*_max_ = 0.932	*l* = −13→13
6585 measured reflections	

### Refinement

**Table:**

Refinement on *F*^2^	Primary atom site location: structure-invariant direct methods
Least-squares matrix: full	Secondary atom site location: difference Fourier map
*R*[*F*^2^ > 2σ(*F*^2^)] = 0.038	Hydrogen site location: difference Fourier map
*wR*(*F*^2^) = 0.107	H-atom parameters constrained
*S* = 1.06	*w* = 1/[σ^2^(*F*_o_^2^) + (0.0598*P*)^2^ + 0.2839*P*] where *P* = (*F*_o_^2^ + 2*F*_c_^2^)/3
3270 reflections	(Δ/σ)_max_ < 0.001
227 parameters	Δρ_max_ = 0.49 e Å^−^^3^
0 restraints	Δρ_min_ = −0.45 e Å^−^^3^

### Special details

**Table:**

Geometry. All esds (except the esd in the dihedral angle between two l.s. planes) are estimated using the full covariance matrix. The cell esds are taken into account individually in the estimation of esds in distances, angles and torsion angles; correlations between esds in cell parameters are only used when they are defined by crystal symmetry. An approximate (isotropic) treatment of cell esds is used for estimating esds involving l.s. planes.
Refinement. Refinement of F^2^ against ALL reflections. The weighted R-factor wR and goodness of fit S are based on F^2^, conventional R-factors R are based on F, with F set to zero for negative F^2^. The threshold expression of F^2^ > 2sigma(F^2^) is used only for calculating R-factors(gt) etc. and is not relevant to the choice of reflections for refinement. R-factors based on F^2^ are statistically about twice as large as those based on F, and R- factors based on ALL data will be even larger.

### Fractional atomic coordinates and isotropic or equivalent isotropic displacement parameters (Å^2^)

**Table:**

	*x*	*y*	*z*	*U*_iso_*/*U*_eq_	
Cl	−0.03805 (7)	0.27602 (6)	−0.01980 (6)	0.04107 (17)	
S	0.16845 (6)	0.39221 (5)	0.42629 (5)	0.02979 (16)	
O1	0.23043 (17)	−0.01506 (14)	0.46155 (13)	0.0285 (3)	
O2	0.04121 (18)	0.47817 (16)	0.34509 (18)	0.0435 (4)	
C1	0.1811 (2)	0.2199 (2)	0.4171 (2)	0.0264 (4)	
C2	0.1373 (2)	0.1876 (2)	0.3117 (2)	0.0267 (4)	
C3	0.0730 (2)	0.2646 (2)	0.1967 (2)	0.0305 (5)	
H3	0.0487	0.3621	0.1712	0.037*	
C4	0.0477 (2)	0.1874 (2)	0.1228 (2)	0.0306 (5)	
C5	0.0854 (2)	0.0415 (2)	0.1569 (2)	0.0310 (5)	
H5	0.0673	−0.0043	0.1024	0.037*	
C6	0.1494 (2)	−0.0362 (2)	0.2703 (2)	0.0288 (4)	
C7	0.1711 (2)	0.0418 (2)	0.3446 (2)	0.0274 (4)	
C8	0.2345 (2)	0.0952 (2)	0.5053 (2)	0.0276 (4)	
C9	0.2953 (2)	0.0527 (2)	0.6299 (2)	0.0279 (4)	
C10	0.2804 (3)	0.1420 (2)	0.7028 (2)	0.0359 (5)	
H10	0.2325	0.2341	0.6711	0.043*	
C11	0.3366 (3)	0.0941 (3)	0.8220 (2)	0.0405 (5)	
H11	0.3266	0.1544	0.8697	0.049*	
C12	0.4074 (3)	−0.0426 (3)	0.8707 (2)	0.0428 (6)	
H12	0.4440	−0.0746	0.9513	0.051*	
C13	0.4234 (3)	−0.1312 (3)	0.7991 (2)	0.0434 (6)	
H13	0.4721	−0.2229	0.8310	0.052*	
C14	0.3674 (3)	−0.0846 (2)	0.6801 (2)	0.0359 (5)	
H14	0.3779	−0.1457	0.6331	0.043*	
C15	0.1942 (3)	−0.1933 (2)	0.3103 (2)	0.0375 (5)	
H15A	0.1356	−0.2332	0.2670	0.056*	
H15B	0.1635	−0.2360	0.4036	0.056*	
H15C	0.3121	−0.2110	0.2857	0.056*	
C16	0.3687 (2)	0.44102 (19)	0.3307 (2)	0.0255 (4)	
C17	0.3857 (3)	0.5151 (2)	0.1978 (2)	0.0384 (5)	
H17	0.2941	0.5382	0.1549	0.046*	
C18	0.5425 (4)	0.5546 (3)	0.1292 (3)	0.0523 (7)	
H18	0.5564	0.6043	0.0394	0.063*	
C19	0.6776 (3)	0.5206 (3)	0.1937 (3)	0.0537 (7)	
H19	0.7824	0.5467	0.1469	0.064*	
C20	0.6586 (3)	0.4486 (3)	0.3261 (3)	0.0479 (6)	
H20	0.7501	0.4268	0.3691	0.058*	
C21	0.5045 (3)	0.4088 (2)	0.3955 (2)	0.0349 (5)	
H21	0.4912	0.3604	0.4855	0.042*	

### Atomic displacement parameters (Å^2^)

**Table:**

	*U*^11^	*U*^22^	*U*^33^	*U*^12^	*U*^13^	*U*^23^
Cl	0.0383 (3)	0.0419 (3)	0.0439 (3)	−0.0030 (2)	−0.0165 (2)	−0.0105 (3)
S	0.0222 (3)	0.0257 (3)	0.0467 (3)	0.00223 (19)	−0.0041 (2)	−0.0209 (2)
O1	0.0335 (7)	0.0224 (7)	0.0312 (8)	−0.0028 (6)	−0.0043 (6)	−0.0114 (6)
O2	0.0248 (7)	0.0356 (9)	0.0799 (12)	0.0119 (6)	−0.0211 (8)	−0.0301 (9)
C1	0.0217 (9)	0.0231 (10)	0.0367 (11)	−0.0007 (8)	−0.0024 (8)	−0.0147 (9)
C2	0.0215 (9)	0.0251 (10)	0.0358 (11)	−0.0021 (8)	−0.0024 (8)	−0.0146 (9)
C3	0.0241 (10)	0.0258 (10)	0.0424 (12)	−0.0010 (8)	−0.0056 (9)	−0.0131 (9)
C4	0.0238 (10)	0.0329 (11)	0.0340 (11)	−0.0039 (8)	−0.0052 (8)	−0.0095 (9)
C5	0.0286 (10)	0.0333 (11)	0.0350 (11)	−0.0070 (9)	−0.0019 (9)	−0.0162 (9)
C6	0.0273 (10)	0.0271 (10)	0.0346 (11)	−0.0046 (8)	−0.0017 (8)	−0.0144 (9)
C7	0.0242 (10)	0.0282 (10)	0.0306 (11)	−0.0041 (8)	−0.0006 (8)	−0.0123 (8)
C8	0.0242 (10)	0.0257 (10)	0.0359 (11)	−0.0038 (8)	0.0006 (8)	−0.0164 (9)
C9	0.0235 (10)	0.0294 (10)	0.0308 (11)	−0.0051 (8)	0.0020 (8)	−0.0127 (9)
C10	0.0424 (12)	0.0294 (11)	0.0377 (12)	−0.0033 (9)	−0.0037 (10)	−0.0150 (10)
C11	0.0482 (14)	0.0449 (13)	0.0358 (13)	−0.0084 (11)	−0.0051 (10)	−0.0220 (11)
C12	0.0461 (14)	0.0468 (14)	0.0372 (13)	−0.0050 (11)	−0.0118 (11)	−0.0133 (11)
C13	0.0469 (14)	0.0369 (13)	0.0453 (14)	0.0067 (11)	−0.0148 (11)	−0.0129 (11)
C14	0.0379 (12)	0.0332 (12)	0.0401 (13)	0.0020 (9)	−0.0064 (10)	−0.0187 (10)
C15	0.0466 (13)	0.0268 (11)	0.0440 (13)	−0.0032 (10)	−0.0076 (10)	−0.0176 (10)
C16	0.0221 (9)	0.0182 (9)	0.0390 (11)	0.0017 (7)	−0.0069 (8)	−0.0135 (8)
C17	0.0424 (13)	0.0303 (11)	0.0418 (13)	0.0059 (10)	−0.0148 (10)	−0.0109 (10)
C18	0.0673 (18)	0.0342 (13)	0.0413 (15)	−0.0070 (12)	0.0066 (13)	−0.0036 (11)
C19	0.0351 (13)	0.0378 (14)	0.080 (2)	−0.0092 (11)	0.0102 (13)	−0.0188 (14)
C20	0.0264 (11)	0.0413 (13)	0.080 (2)	−0.0023 (10)	−0.0134 (12)	−0.0232 (13)
C21	0.0321 (11)	0.0317 (11)	0.0438 (13)	−0.0012 (9)	−0.0144 (10)	−0.0126 (10)

### Geometric parameters (Å, °)

**Table:**

Cl—C4	1.747 (2)	C10—H10	0.9300
S—O2	1.489 (2)	C11—C12	1.382 (3)
S—O2^i^	3.327 (2)	C11—H11	0.9300
S—C1	1.778 (2)	C12—C13	1.378 (3)
S—C16	1.794 (2)	C12—H12	0.9300
O1—C7	1.374 (2)	C13—C14	1.382 (3)
O1—C8	1.381 (2)	C13—H13	0.9300
C1—C8	1.368 (3)	C14—H14	0.9300
C1—C2	1.446 (3)	C15—H15A	0.9600
C2—C7	1.391 (3)	C15—H15B	0.9600
C2—C3	1.397 (3)	C15—H15C	0.9600
C3—C4	1.384 (3)	C16—C17	1.378 (3)
C3—H3	0.9300	C16—C21	1.387 (3)
C4—C5	1.396 (3)	C17—C18	1.388 (3)
C5—C6	1.383 (3)	C17—H17	0.9300
C5—H5	0.9300	C18—C19	1.378 (4)
C6—C7	1.384 (3)	C18—H18	0.9300
C6—C15	1.505 (3)	C19—C20	1.370 (4)
C8—C9	1.460 (3)	C19—H19	0.9300
C9—C14	1.392 (3)	C20—C21	1.372 (3)
C9—C10	1.397 (3)	C20—H20	0.9300
C10—C11	1.384 (3)	C21—H21	0.9300
			
O2—S—C1	105.84 (9)	C12—C11—H11	119.7
O2—S—C16	106.83 (10)	C10—C11—H11	119.7
C1—S—C16	97.45 (9)	C13—C12—C11	119.5 (2)
C7—O1—C8	107.09 (15)	C13—C12—H12	120.3
C8—C1—C2	107.53 (17)	C11—C12—H12	120.3
C8—C1—S	127.27 (16)	C12—C13—C14	120.4 (2)
C2—C1—S	125.20 (15)	C12—C13—H13	119.8
C7—C2—C3	119.26 (18)	C14—C13—H13	119.8
C7—C2—C1	104.90 (18)	C13—C14—C9	120.8 (2)
C3—C2—C1	135.84 (18)	C13—C14—H14	119.6
C4—C3—C2	116.10 (19)	C9—C14—H14	119.6
C4—C3—H3	122.0	C6—C15—H15A	109.5
C2—C3—H3	122.0	C6—C15—H15B	109.5
C3—C4—C5	123.5 (2)	H15A—C15—H15B	109.5
C3—C4—Cl	118.61 (16)	C6—C15—H15C	109.5
C5—C4—Cl	117.89 (16)	H15A—C15—H15C	109.5
C6—C5—C4	121.07 (19)	H15B—C15—H15C	109.5
C6—C5—H5	119.5	C17—C16—C21	120.8 (2)
C4—C5—H5	119.5	C17—C16—S	120.98 (16)
C5—C6—C7	114.86 (18)	C21—C16—S	118.11 (17)
C5—C6—C15	123.06 (18)	C16—C17—C18	118.7 (2)
C7—C6—C15	122.08 (19)	C16—C17—H17	120.7
O1—C7—C6	124.18 (18)	C18—C17—H17	120.7
O1—C7—C2	110.62 (17)	C19—C18—C17	120.3 (2)
C6—C7—C2	125.2 (2)	C19—C18—H18	119.8
C1—C8—O1	109.86 (17)	C17—C18—H18	119.8
C1—C8—C9	135.94 (18)	C20—C19—C18	120.5 (2)
O1—C8—C9	114.20 (17)	C20—C19—H19	119.8
C14—C9—C10	118.4 (2)	C18—C19—H19	119.8
C14—C9—C8	118.49 (18)	C19—C20—C21	120.0 (2)
C10—C9—C8	123.04 (19)	C19—C20—H20	120.0
C11—C10—C9	120.3 (2)	C21—C20—H20	120.0
C11—C10—H10	119.9	C20—C21—C16	119.7 (2)
C9—C10—H10	119.9	C20—C21—H21	120.1
C12—C11—C10	120.6 (2)	C16—C21—H21	120.1
			
O2—S—C1—C8	−156.31 (18)	C2—C1—C8—C9	179.9 (2)
C16—S—C1—C8	93.77 (19)	S—C1—C8—C9	0.6 (4)
O2—S—C1—C2	24.43 (19)	C7—O1—C8—C1	−0.8 (2)
C16—S—C1—C2	−85.48 (18)	C7—O1—C8—C9	−179.96 (15)
C8—C1—C2—C7	−0.9 (2)	C1—C8—C9—C14	−168.4 (2)
S—C1—C2—C7	178.49 (14)	O1—C8—C9—C14	10.4 (3)
C8—C1—C2—C3	178.3 (2)	C1—C8—C9—C10	13.4 (4)
S—C1—C2—C3	−2.3 (3)	O1—C8—C9—C10	−167.76 (18)
C7—C2—C3—C4	0.0 (3)	C14—C9—C10—C11	0.1 (3)
C1—C2—C3—C4	−179.1 (2)	C8—C9—C10—C11	178.25 (19)
C2—C3—C4—C5	−1.1 (3)	C9—C10—C11—C12	−0.3 (3)
C2—C3—C4—Cl	178.18 (14)	C10—C11—C12—C13	0.6 (4)
C3—C4—C5—C6	1.1 (3)	C11—C12—C13—C14	−0.8 (4)
Cl—C4—C5—C6	−178.25 (15)	C12—C13—C14—C9	0.6 (4)
C4—C5—C6—C7	0.2 (3)	C10—C9—C14—C13	−0.2 (3)
C4—C5—C6—C15	−179.2 (2)	C8—C9—C14—C13	−178.5 (2)
C8—O1—C7—C6	179.90 (18)	O2—S—C16—C17	−15.11 (19)
C8—O1—C7—C2	0.3 (2)	C1—S—C16—C17	93.99 (18)
C5—C6—C7—O1	178.93 (17)	O2—S—C16—C21	161.49 (15)
C15—C6—C7—O1	−1.7 (3)	C1—S—C16—C21	−89.42 (17)
C5—C6—C7—C2	−1.5 (3)	C21—C16—C17—C18	1.2 (3)
C15—C6—C7—C2	177.9 (2)	S—C16—C17—C18	177.74 (17)
C3—C2—C7—O1	−178.94 (16)	C16—C17—C18—C19	−0.3 (4)
C1—C2—C7—O1	0.4 (2)	C17—C18—C19—C20	−0.6 (4)
C3—C2—C7—C6	1.4 (3)	C18—C19—C20—C21	0.6 (4)
C1—C2—C7—C6	−179.25 (19)	C19—C20—C21—C16	0.4 (3)
C2—C1—C8—O1	1.1 (2)	C17—C16—C21—C20	−1.3 (3)
S—C1—C8—O1	−178.27 (13)	S—C16—C21—C20	−177.88 (17)

Symmetry codes: (i) −*x*, −*y*+1, −*z*+1.

### Hydrogen-bond geometry (Å, °)

**Table:**

*D*—H···*A*	*D*—H	H···*A*	*D*···*A*	*D*—H···*A*
C19—H19···Cl^ii^	0.93	2.78	3.653 (3)	157
C20—H20···O2^iii^	0.93	2.47	3.261 (3)	144

Symmetry codes: (ii) −*x*+1, −*y*+1, −*z*; (iii) *x*+1, *y*, *z*.

## References

[bb1] Allen, F. H., Baalham, C. A., Lommerse, J. P. M. & Raithby, P. R. (1998). *Acta Cryst.* B**54**, 320–329.

[bb2] Brandenburg, K. (1998). *DIAMOND* Crystal Impact GbR, Bonn, Germany.

[bb3] Bruker (2001). *SAINT* and *SMART* Bruker AXS Inc., Madison, Wisconsin, USA.

[bb4] Choi, H. D., Seo, P. J., Son, B. W. & Lee, U. (2007). *Acta Cryst.* E**63**, o1291–o1292.

[bb5] Choi, H. D., Seo, P. J., Son, B. W. & Lee, U. (2008*a*). *Acta Cryst.* E**64**, o1190. 10.1107/S1600536808015699 PMC2961728 21202832

[bb6] Choi, H. D., Seo, P. J., Son, B. W. & Lee, U. (2008*b*). *Acta Cryst.* E**64**, o1061. 10.1107/S1600536808013706 PMC2961388 21202580

[bb7] Farrugia, L. J. (1997). *J. Appl. Cryst.* **30**, 565.

[bb8] Sheldrick, G. M. (1999). *SADABS* University of Göttingen, Germany.

[bb9] Sheldrick, G. M. (2008). *Acta Cryst.* A**64**, 112–122. 10.1107/S0108767307043930 18156677

